# Extracellular Vesicles as Mediators of Cellular Cross Talk in the Lung Microenvironment

**DOI:** 10.3389/fmed.2020.00326

**Published:** 2020-08-04

**Authors:** Sabine Bartel, Jessy Deshane, Tom Wilkinson, Susanne Gabrielsson

**Affiliations:** ^1^Department of Pathology and Medical Biology, GRIAC Research Institute, University of Groningen, University Medical Center Groningen, Groningen, Netherlands; ^2^Pulmonary Allergy and Critical Care Medicine, University of Alabama at Birmingham, Birmingham, AL, United States; ^3^Clinical and Experimental Science, Faculty of Medicine, University of Southampton, Southampton, United Kingdom; ^4^Division of Immunology and Allergy, Department of Medicine Solna, Karolinska Institute, Stockholm, Sweden; ^5^Department of Clinical Immunology and Transfusion Medicine, Karolinska University Hospital, Stockholm, Sweden

**Keywords:** extracellular vesicles, exosomes, asthma, chronic obstructive pulmonary disease, sarcoidosis, microRNA

## Abstract

The human lung is a complex tissue subdivided into several regions that differ in size, function, and resident cell types. Despite years of intensive research, we still do not fully understand the cross talk between these different regions and diverse cell populations in the lung and how this is altered in the development of chronic respiratory disease. The discovery of extracellular vesicles (EVs), small membrane vesicles released from cells for intercellular communication, has added another layer of complexity to cellular cross talk in the complex lung microenvironment. EVs from patients with chronic obstructive pulmonary disease, asthma, or sarcoidosis have been shown to carry microRNAs, proteins, and lipids that may contribute to inflammation or tissue degeneration. Here, we summarize the contribution of these small vesicles in the interplay of several different cell types in the lung microenvironment, with a focus on the development of chronic respiratory diseases. Although there are already many studies demonstrating the adverse effects of EVs in the diseased lung, we still have substantial knowledge gaps regarding the concrete role of EV involvement in lung disease, which should be addressed in future studies.

## Introduction

Extracellular vesicles (EVs) are bilayered lipid membrane vesicles that are released by every cell type in our body. There are several different EV-subclasses with distinct markers (1). Roughly, EVs are subcategorized according to size and cellular origin into exosomes (40–150 nm) derived from the budding of the early-endosomal membrane and microvesicles (>1 μm) shed from the plasma membrane (2). Of note, the common EV isolation methods such as ultracentrifugation, size-exclusion chromatography, and density gradients separate EVs based on size and/or density, and isolate a mix of different EV populations, thus, we will here only refer to EVs but not distinct subclasses (3).

Most EVs have been shown to contain RNAs [including small non-coding RNA, such as microRNA [miRNA]], DNA, lipids, and proteins (4, 5). The scientific interest in EVs exploded when it became evident that they can functionally transfer molecules between cells (6, 7). Furthermore, additional interest in their role has been driven by observations that EV uptake might be at least partly receptor-mediated and therewith cell-specific (8). EVs can also exert their function by sticking to the surface of recipient cells. Dendritic cells (DCs) use EVs to present novel antigens via the major histocompatibility complex (MHC) II on the EV surface (9). Although there are strong hints for EVs being functionally involved in cellular communication, it is not clear whether this always includes cell-to-cell transfer of molecules, as the detailed molecular processes regulating EV uptake are not fully understood (10).

We here aim to shed light on the functional role of EV-mediated communication between distinct cell types in a complex tissue—the human lung. As the lung is a barrier organ to the outside, it requires constant cross talk between both structural and immune cells to restore homeostasis and protect the body from external pathogens. Hereto, we will discuss current evidence on distinct EV-mediated pulmonary communication, while also discussing aberrations thereof in chronic lung diseases, such as asthma, chronic obstructive pulmonary disease (COPD), and sarcoidosis.

## The Lung Microenvironment in Homeostasis and Disease

The human lung harbors a plethora of different structural cells (11) that, in order to maintain tissue homeostasis and defense against external pathogens, are in constant cross talk with each other and with immune cells. There is substantial knowledge of the nature of receptor-ligand interactions, different growth factors, and cytokines; however, these do not fully explain all known molecular events. Thus, EVs might represent a missing link in cellular communication in the lung microenvironment, and understanding their role more completely could help explain mechanisms driving chronic lung disease.

Asthma and COPD are characterized by airway obstruction and chronic airway inflammation. In asthma, depending on the subtype, the inflammation can be allergic, eosinophilic, and Th2-prone, or non-allergic neutrophilic and Th17-based (12). COPD, on the other hand, is characterized by a complex inflammatory environment, coordinated by aging and dysregulated immune system (13, 14), driven by responses to inhaled pollutants, predominantly tobacco smoke. Furthermore, COPD is a heterogeneous condition with differing contributions of small airways disease and emphysematous changes in individuals (15, 16). Progression of these pathologies leads to lung function deterioration over time and systemic manifestations associated with significant multi-morbidity (17).

Sarcoidosis is a systemic inflammatory disease that can display multiple organ system manifestations, but it predominantly affects the lung with non-necrotizing granulomas that contain epithelial cells, macrophages, and CD4^+^ T cells of mainly Th1, and Th17 types (18). The etiology is still unclear, but the disease has both genetic and environmental associations. More than half of the patients show respiratory symptoms, including dyspnea, cough, and chest pain. Spontaneous remission occurs in two-thirds of patients, but some develop chronic disease that may result in fibrosis and respiratory failure.

## Extracellular Vesicles from Unknown Cellular Origin

Several studies report changes in the molecular content of EVs isolated from bronchoalveolar lavage fluid (BALF) in asthma (19–21), COPD (22), and sarcoidosis (23–26).

Thereby, all three diseases have been associated with an aberrant miRNA content of BALF EVs compared with healthy controls (20, 22, 26). However, these miRNAs have already been reported to be dysregulated in other diseases, meaning that they are unlikely suitable as a biomarker, unless combined with other markers. EVs from both patients with asthma and sarcoidosis (19, 23) contain enzymes for the biogenesis of pro-inflammatory leukotrienes (LTs) and have pro-inflammatory effects when applied to healthy cells. BALF EVs from patients with idiopathic pulmonary fibrosis, a progressive fibrotic lung disease, have been shown to enhance myofibroblast differentiation via Wingless/Integrase I (WNT) signaling (27).

One could thus speculate that an altered molecular content of EVs might be functionally involved in disease pathogenesis. However, as those studies analyze the EVs of patients with established disease, the changes in EV content could also simply reflect a different cellular composition and function in a diseased lung. Additionally, BALF represents a mixture of EVs from several different cell types, hampering conclusions about cross talk of single cells in the lung. Recently, it has been shown that EVs can travel through hydrogels composed of extracellular matrix (28), indicating that they will be able to move among cells in the lung mesenchyme as well.

In the following review, we will discuss *in vitro*/*ex vivo* studies that specifically studied the EV-mediated interaction of resident lung cells.

## Structural Cells

### Airway Epithelial Cells

The airway epithelium plays a pivotal role in the lung, most likely due to its strategic position at the interface between the body and the outside world. Next to forming a tight physical barrier, it has a strong influence on regulating underlying immunity and is important for host defense against pathogens (29). This function is achieved by the release of antimicrobial peptides, cytokines, chemokines, and alarmins. In particular, alarmins, such as interleukin (IL) 33, thymic stromal lymphopoietin, and IL-25, have gained a lot of scientific attention, as they activate DC and induce a subsequent Th2 polarization of naïve T cells that seems pivotal in asthma development (30, 31). On the other hand, the airway epithelium communicates to structural cells, such as fibroblasts (32, 33) and smooth muscle cells (34) via secretion of cytokines and growth factors. It seems logical, yet much less well-understood, that the epithelium also uses EVs as tools to communicate within the lung.

As can be seen in the interaction matrix in Table 1, EVs from the airway epithelium have been reported to have functional effects on other epithelial cells, fibroblasts, and also macrophages, DCs, and neutrophils. EVs from a human bronchial epithelial cell line (BEAS-2B) treated with IL-13 had pro-inflammatory effects in mice and increased macrophage chemotaxis (35). Recently, it has been shown that primary human bronchial epithelial cells cultured at the air–liquid interface release EVs to the apical (air-exposed) and basal (toward the culture medium) side (36). Upon IL-13 stimulation, mimicking the development of an asthmatic epithelium, these EVs contain lower levels of miRNAs miR-92b, miR-210, and miR-34a. The change in miRNA levels was predicted to influence DC and to promote Th2 differentiation (Table 1). Of note, treating bronchial epithelial cells with cigarette smoke extract (CSE) to model the early development of COPD increased the levels of miR-210 in EVs (37). miR-210 was able to control autophagy processes and induced myofibroblast differentiation. Thus, the EV-mediated dysregulation of myofibroblast development could be involved in remodeling in COPD.

**Table 1:** EV Interaction matrix in the lung.

		**EV Recipient**									
		**Airway epithelium**	**Smooth muscle**	**Fibroblasts**	**Macrophages**	**Dendritic cells**	**Eosinophils**	**Neutrophils**	**T-cells**	**B-cells**	**Unknown**
**EV Donors**	Airway epithelium	- Pro-inflammatory (TN-C)^38^ –Innate defense against influenza (MUC1,4,16)^39^ –IL-8 and MMP1 secretion ↑ (cleaved CCN1)^40^		Myofibroblast differentiation ↑ (miR-210↑)^37^	*Chemotaxis* ↑*^35^* pro-inflammatory (TN-C)^38^	Th2 polarization (miRNA-92b, miR-210 and miR-34a↓)^36^		Chemotaxis ↑ (S100 A12)^42^			
	Smooth muscle										
	Fibroblasts	Proliferation ↑ (TGF-β2↓)^45^		Inhibition of myofibroblast differentiation (PGE_2_)^46^							
	Macrophages	- Dampening of inflammation (SOCS1, SOCS3)^52, 53^ –proinflammatory IL-6 and TNF-α↑^55^ –*ICAM1 expression and cytokine secretion ↑ (TNFα)^61^*	Enzymes for biosynthesis of leukotrienes^64^		-Differentiation of monocytes into macrophages↑(miR-223)^57^ –after bacterial infection: TNF-α secretion ↑ (bacterial PAMPs)^60^			- Migration (chemotactic eicosanoids)^64^ –TNF-α secretion ↑ (bacterial PAMPs)^60^	Ag presentation via MHCII and Th2 polarization^58^		Gelatinolytic and collagenolytic activity (MMP-14)^56^
	Dendritic cells		Enzymes for biosynthesis of leukotrienes^64^					Migration (chemotactic eicosanoids)^64^	- Th2 polarization (OX40L↑)^63^ –Ag- presentation and Th2 polarization^58^		
	Eosinophils	Apoptosis ↑(mechanism unknown)^65^	Proliferation ↑(mechanism unknown)^65^				Autoregulation (nitric oxide, reactive oxygen species)^66^ Chemotaxis^66^				
	Neutrophils										Destruction of collagen fibers (emphysema) (NE)^67^
	T-cells										
	B-cells								Ag presentation via MHCII and Th2 polarization^70^		

*
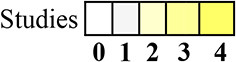
Cellular interaction in the lung via EVs from donor cells (rows) to recipient cells (columns) and responsible molecule in brackets (if known); Reference in superscript, italic means murine study; blue structural cell types, green: immune cell types*.

Airway epithelial cells from patients with asthma release more tenascin-C (TN-C)-carrying EVs upon rhinovirus infection than healthy ones (38). These EVs induced pro-inflammatory responses in macrophages and a bronchial epithelial cell line. However, the latter could not be reduced by decreasing the levels of TN-C on the EVs, suggesting that it is not the only important player within the EVs. Human tracheobronchial epithelial cells cultured *in vitro* secreted EVs with membrane-tethered mucins, including MUC1, MUC4, and MUC16 (39). These were found to directly neutralize influenza, contributing to the innate defense of the airway epithelium. Furthermore, EV-associated cleaved *Cellular Communication Network Factor 1* (CCN1) was able to activate the secretion of IL-8 and *Matrix metalloprotease 1* (MMP-1) from epithelial cells. CSE exposure of epithelial cells induced the production of EVs containing CCN1 *in vitro* (40). Dysregulation in the production of MMP has been associated with lung matrix destruction and small airways disease in COPD (41). Thus, the delivery of MMPs through EVs may be involved in the development of emphysema.

Airway epithelial cells from cystic fibrosis patients secreted more EVs than cells from healthy controls when cultured in air–liquid interface (42). They also had a different protein cargo and increased chemotaxis of neutrophils to the airways via S100 A12 (42). Of note, as cystic fibrosis is a monogenetic disease characterized by mutations in the *Cystic Fibrosis Transmembrane Conductance Regulator (CFTR)*, one could speculate that host genetics plays a role in the secretion and content of EVs. However, *CFTR* mutations largely impact the function and differentiation of the epithelium due to the diminished secretion of Cl^−^ anions and formation of sticky mucus on the surface (43). This, in turn, could affect EV secretion. Thus, further studies are needed to pinpoint the effect of genetics vs. the environment.

In order to determine the relative contributions of epithelial EVs in the airways, Pua et al. (44) analyzed airway lining fluid (bronchial washes) of mice and showed by flow cytometry that around 80% of EVs were derived from epithelial cells and had a similar miRNA signature. After allergen-challenge, the presence of 12 immune-related miRNAs (i.e., miR-142a and miR-223) increased 2-fold compared with healthy control mice. However, this does not necessarily imply that the airway epithelium produces the most EVs in the lung microenvironment, as the majority of EVs from other cell types might be held back by the physical epithelial barrier and, thus, do not reach the airway lining fluid.

### Fibroblasts, Mesenchymal Stem/Stromal Cells, and Smooth Muscle Cells

Fibroblasts and smooth muscle cells play an important role in lung homeostasis and disease. They regulate epithelial cell functions through the secretion of growth factors, cytokines, chemokines, but also EVs. In turn, lung diseases such as COPD, idiopathic pulmonary fibrosis, and asthma are characterized by different extents of hyperplasia of both cell types, called airway remodeling. EVs from bronchial fibroblasts have been shown to modulate epithelial cell proliferation by TGF-β2-dependent mechanisms (45). Furthermore, activated human fibroblasts also inhibit the myofibroblast differentiation of other fibroblasts via EV-enclosed Prostaglandin E2 (PGE_2_) (46). Also, whole mitochondria can be transferred between cells via EVs (47–49). In response to intracellular oxidative stress, mesenchymal stromal/stem cells shuttle depolarized mitochondria by mitophagy within EVs to be engulfed by macrophages. This contributes to the alteration in cellular bioenergetics and function in the recipient cells but can also constitute danger signals (47–49). To our knowledge, there is no study investigating the EV secretion of airway smooth muscle cells yet, but according to Table 1, this cell type seems to be influenced by EVs derived from immune cells.

### Immune Cells

As mentioned earlier, the lung forms a barrier to the outside world. To avoid invasion of pathogens, innate immune cells such as macrophages and DCs are continuously patrolling our airways and can call in eosinophils and neutrophils or adaptive immune cells if needed (11).

### Macrophages

There is increasing appreciation for macrophage plasticity and dichotomy: alveolar macrophages (AMs) suppress, whereas recruited monocyte-derived macrophages play largely pathogenic roles in asthma (50, 51). Two studies have found that EVs derived from AMs contain suppressor of cytokine signaling (SOCS)-1 and SOCS-3 proteins (52, 53). Treatment of epithelial cells with these EVs decreased cytokine signaling through JAK–STAT activation. Of note, a decreased concentration of SOCS proteins has been found in the BAL fluid of smokers (54). Dysregulation of the delivery of SOCS proteins through EV could, therefore, be an important mechanism in the derangement of cytokine signaling in chronic airway inflammation. AMs are directly exposed to environmental antigens and particulate matter (PM). Exposure to PM induces the release of EVs in a dose-dependent manner, and the PM-induced EVs exert a pro-inflammatory phenotype on pulmonary epithelial cells, resulting in the release of the pro-inflammatory cytokines IL-6 and tumor necrosis factor α (TNF-α) (55). EVs derived from CSE-exposed macrophages have been shown to contain MMP-14 with gelatinolytic and collagenolytic activity and might, therefore, be involved in emphysema development in COPD (56).

**Figure 1:**
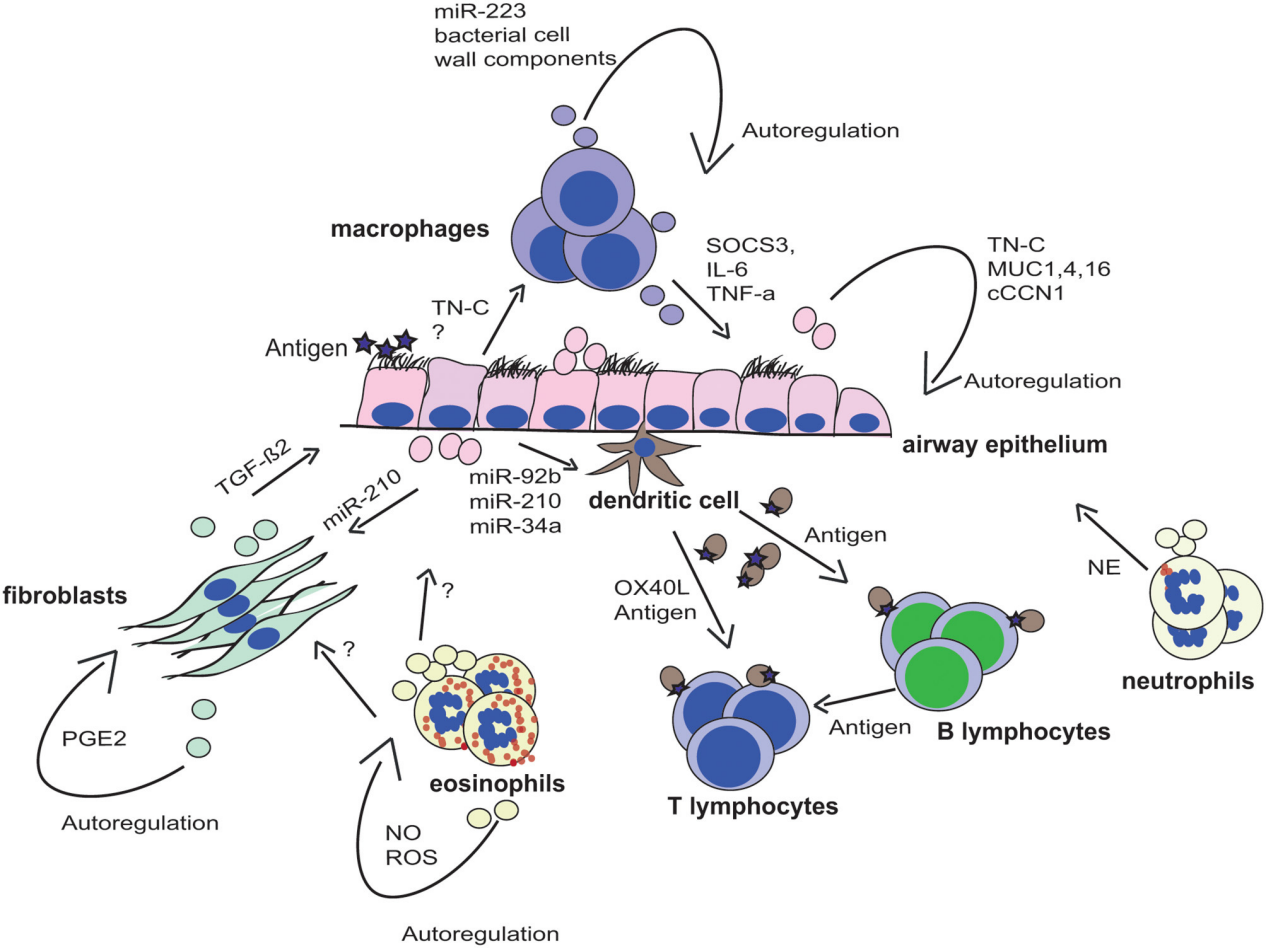
EV-mediated cross talk in the lung microenvironment. The lung microenvironment is characterized by a complex cross talk of several different cell types. Secreted extracellular vesicles (EV) from these cells have been shown to play critical roles in the tissue homeostasis or the development of chronic respiratory disease due to the transfer of molecules to other cell types. EVs are displayed in the respective color of the parent cell, and transferred molecules are indicated next to the arrows.

EVs produced from a differentiated monocyte cell line *in vitro* induced the differentiation of naive monocytes into macrophages that was dependent on miR-223 (57). The accumulation of dysfunctional macrophages is characteristic of the COPD lung, and EV-miR-223 may provide an amplification loop for monocyte differentiation (Figure 1). Macrophage and DC-derived EVs also contain MHC class II and co-stimulatory molecules (58), providing a route for antigen presentation and immune activation in the lung. A key pathway in the production of inflammatory cytokines by macrophages is through Toll-like receptor stimulation by pathogen-associated molecular patterns (PAMPs) (59). A study found macrophages infected with bacteria to produce EVs containing bacterial cell wall components. These EVs were shown to stimulate the release of TNF-α by macrophages and neutrophils in a mouse model (60). Other studies have shown the direct transport of TNF-α in EVs from macrophages and epithelial cells upon LPS stimulation (61).

#### Dendritic Cells

Most knowledge on lung-resident DCs is based on the development of asthma, where they play a pivotal role in establishing an allergen-specific Th2 response in the airways after stimulation with epithelial alarmins (62). A recent study showed that DCs stimulated *in vitro* with the epithelial alarmin thymic stromal lymphopoietin secrete EVs expressing OX40L on their surface, via which they stimulate the proliferation of CD4 T cells and Th2 differentiation (63). Furthermore, EVs secreted by monocyte-derived DCs purified from human airways express HLA-DR, MHC-I molecules, CD63, CD86, and CD54, suggesting their potential to cross-present antigen-loaded MHC molecules mediating co-stimulation (21). DC-derived EVs also contribute enzymes for biosynthesis of LTs, key pro-inflammatory mediators important in the pathogenesis of asthma, to smooth muscle cells. Additionally, these EVs produced chemotactic eicosanoids and induced granulocyte migration (64). Of note, LTs and their enzymes have also been found in BALF EVs of patients with asthma (19) and sarcoidosis (25). The importance of LTs in asthma is established, but the role in sarcoidosis is unknown and needs further investigation.

### Eosinophils

Eosinophils infiltrate into the lung during the development of asthma and influence several lung-resident cells via EVs (Table 1, Figure 1). Accordingly, eosinophil EVs of patients with asthma induce epithelial cell apoptosis and smooth muscle cell proliferation, both important aspects of asthma pathogenesis (65). Eosinophil-derived EVs also autoregulate themselves in asthma by producing nitric oxide and reactive oxygen species (66) (Figure 1). Moreover, these EVs can act as a chemotactic factor for eosinophils due to expression of adhesion molecules, such as ICAM-1 and integrin α2 (66).

### Neutrophils

Neutrophilic infiltration into the lung is a major characteristic of inflammation caused by cigarette smoking and COPD but is also sometimes observed in non-allergic asthma. A recent study has found neutrophil elastase (NE) in EVs from activated human neutrophils, and those were shown to degrade collagen fibers and induce emphysema development in mice (67). This could be a crucial mechanism in the development of emphysema and should, thus, be further investigated.

### Myeloid-Regulatory Cells and T and B cells

EV-enclosed mitochondria from regulatory myeloid-lineage cells are internalized by CD4^+^ T lymphocytes (49). Furthermore, functional mitochondria within the EVs produce reactive oxygen species, and the transferred mitochondrial components merge with the mitochondrial network of the recipient T cells in asthmatics (49). These regulatory myeloid cells have been shown to modulate T-cell proliferation in persons with asthma (68), and hence, the transfer of mitochondria within EVs may facilitate antigen-presentation and T-cell activation.

T cells produce Th2 cytokines when stimulated with B-cell derived EVs loaded with peptide-loaded MHC-II isolated from patients with birch pollen allergy (69). Similarly, in allergic skin diseases, EVs transfer antigens activating immune responses in B or T cells (69, 70). To our knowledge, there is no study investigating the functional effect of T-cell-derived EVs on lung cells specifically, but the regulations of the immune response might be similar to other tissues.

## Concluding Remarks

Although we have some knowledge on EVs in the lung microenvironment (Figure 1, Table 1), we still do not completely understand their role in the development of respiratory disease. Most of the studies discussed here focus on EVs from isolated cell cultures or a mixture of EVs isolated from human body fluids, although there is still little knowledge on EV-mediated communication in complex cellular interactions in tissues *in vivo*. Furthermore, it is not clear if alterations in EV content are a cause or a consequence of disease. Table 1 represents an interaction matrix of EVs between lung cells, and it clearly shows that the most studied EV-mediated interactions are the cross talk of macrophages with the airway epithelium and vice versa. Generally, it seems like there is EV-mediated cross talk between structural and innate immune cells, but there still are a lot of missing links that are yet to be established. For example, although there are several reports showing that EVs play a role in the interaction of antigen-presenting cells with adaptive immune cells, there has been no study on the effect of EVs derived from activated T or B cells on (structural) lung cells. As many lung diseases are characterized by chronic inflammation, this would certainly be of importance in the future. It also becomes evident that we often do not know the EV donor and/or recipient cell, as EVs have been isolated from body fluids such as BALF, and identifying EV internalizing target cells remains difficult, especially *in vivo*. Knowledge about, that is, surface receptors involved in the (supposedly) specific targeting process could lead to them being used in future therapy. Thus, future studies using i*n vivo* models and three-dimensional cultures are urgently needed to further decipher the reciprocal cross talk. Furthermore, most studies either investigate the EV-associated protein or non-coding RNA content but seldom both, even if the molecules most likely act together. Comprehensive profiling of distinct EV populations, including RNA-seq, proteomics, and lipidomics of the same EVs, will help to gain further insight in their role in the development of lung diseases and will identify their potential as biomarker or even as therapy for respiratory disease.

## Author Contributions

SB, JD, TW, and SG screened the current literature for relevant publications and wrote the respective sections in this manuscript. SB developed the figure. All authors contributed to the final editing and conclusion of the manuscript.

## Conflict of Interest

SG holds a patent on exosome-based cancer therapy and is a member of the Scientific Advisory Board of Anjarium Biosciences, Switzerland. The remaining authors declare that the research was conducted in the absence of any commercial or financial relationships that could be construed as a potential conflict of interest.
